# A 7-min halftime jog mitigated the reduction in sprint performance for the initial 15-min of the second half in a simulated football match

**DOI:** 10.1371/journal.pone.0270898

**Published:** 2022-07-19

**Authors:** Sooil Bang, Jihong Park

**Affiliations:** 1 Athletic Training Laboratory, Graduate School of Physical Education, Kyung Hee University, Yongin, Korea; 2 Department of Sports Medicine, Athletic Training Laboratory, Kyung Hee University, Yongin, Korea; Tokyo Joshi Ika Daigaku Toyo Igaku Kenkyujo Clinic, JAPAN

## Abstract

This study compared the effects of a 7-min shuttle jog during halftime to a control condition (seated rest) on subsequent athletic performance and lower-leg temperature in the second half. Eighteen male football players (22 years, 179 cm, 70 kg, 10 years of athletic career) randomly performed a 20-m shuttle jog (at an intensity of 70% of heart rate maximum) and a seated rest (sitting on a bench) during halftime in two separate sessions. A 5-min football simulation protocol consisting of football-specific activities (jumping, sprinting, kicking, passing, and dribbling at various intensities and distances) was repeated nine times to mimic the first and second half of a football match. Athletic performance (maximal vertical jump height, 20-m sprint time, and the Arrowhead agility test time) recorded during a 15-min period were averaged to represent each time point (first half: T1 to T3; second half: T4 to T6). Lower-leg skin and muscle (using the insulation disk technique) temperature was recorded before and after the first and second half. There was no condition effect over time in maximal vertical jump: F_5,187_ = 0.53, *p* = 0.75, Arrowhead agility test time: F_5,187_ = 1.25, *p* = 0.29, and lower-leg temperature (skin: F_3,119_ = 1.40, *p* = 0.25; muscle: F_3,119_ = 1.08, *p* = 0.36). The 20-m sprint time between conditions during the initial 15-min of the second half was different (condition × time: F_5,187_ = 2.42, *p* = 0.04) that subjects who performed the shuttle jog ran 0.09 sec faster (3.08 sec, *p* = 0.002, ES = 0.68), as compared with those who did the seated rest (3.17 sec). The results of our study confirmed that a decremental effect of the static rest on sprinting performance during the initial period of the second halftime can be attenuated by a halftime warm-up.

## Introduction

A deterioration in athletic performance during the second half, as compared with the first half in football matches is common. For example, professional footballers have shown a decline in the total distance covered [1] and the distance covered by high-intensity running [2]. Within the second half, the first 15-min is the time that players’ physical performance is the lowest [3–5]. Since players’ performance generally improves afterwards, this performance decrement during the initial phase of the second half is thought to be from the lack of recovery from the first half and/or preparation for high-intensity activities in the second half. Therefore, there is a need for halftime conditioning strategies, which help not only to enhance recovery from fatigue but also to be ready for the second half [6, 7].

Traditional static halftime rest (e.g., sitting on a bench) could change the body’s regulatory systems (e.g., autonomic nervous, thermoregulatory, and cardiovascular), resulting in a reduction of the core body and working muscle temperature [8]. For example, a 10-min passive rest after a warm-up activity can result in a 0.4°C decrease in body temperature, which could further lead to a 13% reduction in maximal vertical jump capacity [9]. Therefore, taking a passive rest during halftime is not considered as a recommended halftime strategy to prepare for the subsequent performance in the second half in terms of maintaining the “warmed-up body”. To preserve exercise-induced heat, halftime conditioning strategies such as cycling [10], plyometric and agility exercise [11], football-specific activity [12], and jogging [8, 13] have previously been tested. Common endpoints to determine the effectiveness of such halftime activities include body temperature (core [12] and muscle [14]) and athletic performance (sprint time [10, 11] and maximal vertical jump height [13]).

While the aforementioned studies reported an advantage in favour of warming-up during halftime, as compared with a passive rest, a couple of limitations still need to be addressed. First, the previous halftime warm-up strategies [8, 13] were examined in actual football matches. Although the results of these filed studies are advantageous for ecological validity, the sources of variation among subjects (e.g., movements or distance covered) are potential for data heterogeneity; thus, the results might be confounded. Although there have been studies [10, 12, 14, 15] using simulation protocols across interventions, the protocols were not consisted of football-related running and kicking activities [10, 15] or the effectiveness was examined in relatively small samples (n = 7 [12]; n = 10 [15]; n = 10 [14]; n = 13 [10]). Recently, a 90-min long simulated football match (a 5-min of football simulation protocol: FSP ×9 to mimic the first and second halves) with a fixed distance and intensity has been introduced and validated [3]. The recorded average heart rate (163 bpm) and energy expenditure (1,227 kcal) during the simulated football match fell within the typical ranges of real-football matches (heart rate: 150 and 175 bpm [16]; calorie expenditure (1,200 to 1,500 kcal) [17]). Distance covered by running without ball possession in this simulation protocol (maximal: 720-m; submaximal 1,514-m) was also similar to an actual football match (maximal: 542 ± 214-m; submaximal 1,590 ± 488-m) [18].

Therefore, the purpose of this study was to quantify the effect of a halftime shuttle jog and seated rest on the second half performance in collegiate footballers. For the objective comparisons (without possible confounders), the previously validated FSP [3] was employed using a within-subject design. The endpoints were athletic performance (maximal vertical jump height, 20-m sprint time, and Arrowhead agility test time performed during the football simulation), lower-leg temperature (skin and muscle), and heart rate during a 90-min simulated football matches. A previous study [8] observed a decrement in core and muscle (quadriceps) temperature with a static rest during halftime, which further impaired sprinting performance. Another study [13] reported that a halftime shuttle jog prevented sprinting and jumping performance deterioration in the initial period of the second half. According to the previous data [8, 13], we hypothesised that players who performed a shuttle jog would show better athletic performance and higher lower-leg temperature than those who experienced a seated rest.

## Materials and methods

### Study design and experimental approach

A two-way (condition × time) crossover field study with repeated measures on time was used. Subjects randomly performed one of the two different conditioning strategies (shuttle jog or seated rest) during halftime each session. The random order was generated by a spreadsheet software. Dependent measurements were athletic performance (maximal vertical jump height, 20-m sprint time, and Arrowhead agility test time), lower-leg temperature (skin and muscle), and heart rate.

**Fig 1:**
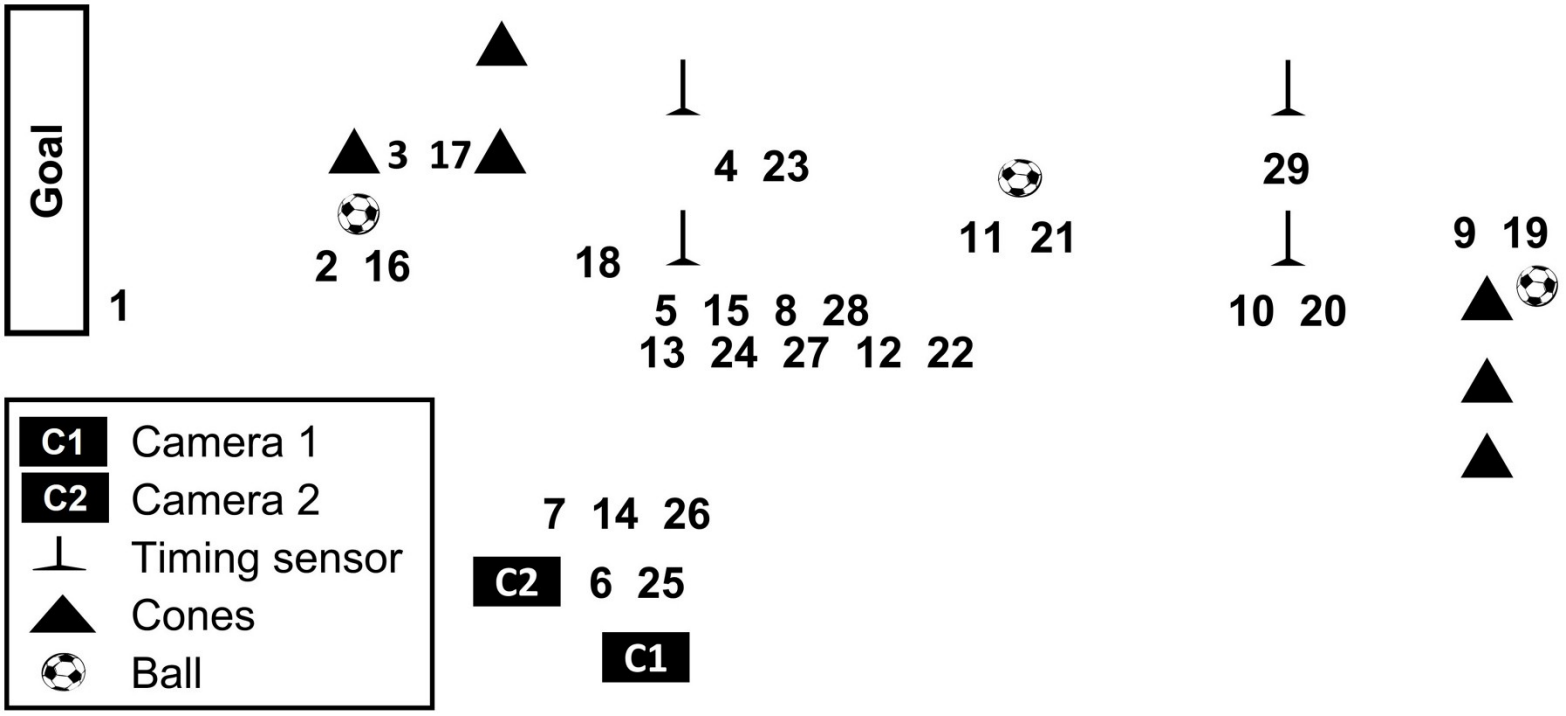
Football simulation protocol. This protocol was repeated three times during each time point. The protocol was to follow the numerical order as follows (1: 10-m run, 2: 10-m short pass ×2, 3: 10-m jog, 4: 40-m Arrowhead agility test, 5: 10-m run, 6: maximal vertical jump ×2, 7: 10-m side step, 8: 20-m walk, 9: 10-m dribble, 10: 10-m walk, 11: 30-m long kick ×2, 12: 10-m run, 13: 10-m back step, 14: 10-m side step, 15: 10-m jog, 16: 10-m short pass ×2, 17: 10-m jog, 18: 20-m sprint, 19: 10-m dribble, 20: walk, 21: 30-m long kick ×2, 22: 10-mjog, 23: 40-m Arrowhead agility test, 24: 10-m back step, 25: maximal vertical jump ×2, 26: 10-m side step, 27: jog, 28: 10-m walk, 29: 20-m sprint).

A 5-min FSP [3] (Fig 1) consisting of football-related activities (a full videoclip is available at https://www.youtube.com/watch?v=9FgWbWMWAa0) were repeated nine times to make up each half of a football match. The athletic performance and heart rate data recorded during the three consecutive FSPs were combined (a total time of 15-min); thus six-time points (first half: T1 through T3; second half: T4 through T6) were analysed and compared. The lower-leg temperatures were measured before and after the first and second half (four time points).

Subjects visited the football pitch (regular-sized natural grass) same time of day on Two different sessions, each 72-h apart. Subjects were asked to maintain their normal diet and wear the same socks and football stud throughout the experimental period and allowed to consume 1.5 L of water during each session (500 mL during each of the first and second halves and halftime). Data were not collected on rainy days. Air temperature and relative humidity were recorded with a digital thermometer (Kestrel Drop, Nielsen-Kellerman Co., Boothwyn, PA, USA) each session and were not different throughout the data collection period (air temperature: t = 0.15, *p* = 0.87; relative humidity: t = –0 .52, *p* = 0.60). The average values of air temperature and relative humidity during the data collection period were 17.5 ± 4.6°C and 40.2 ± 15.9%, respectively.

### Participants

Eighteen male elite collegiate footballers (age: 22 ± 1 years; height: 179 ± 4 cm; mass: 70 ± 5 kg) volunteered for this study. All subjects had to be registered in the Korea Football Association (years of training: 10 ± 1 years) and football trained at least six years. Subjects were excluded if they had any musculoskeletal lower-extremity injury in the past six months, history of back or lower-extremity surgery, or neuromuscular disorders. All subjects read the study procedures, approved by the university’s institutional review board (protocol #: KHSIRB-18-012), and gave written informed consent prior to taking part in the study.

### Testing procedures

**Fig 2:**
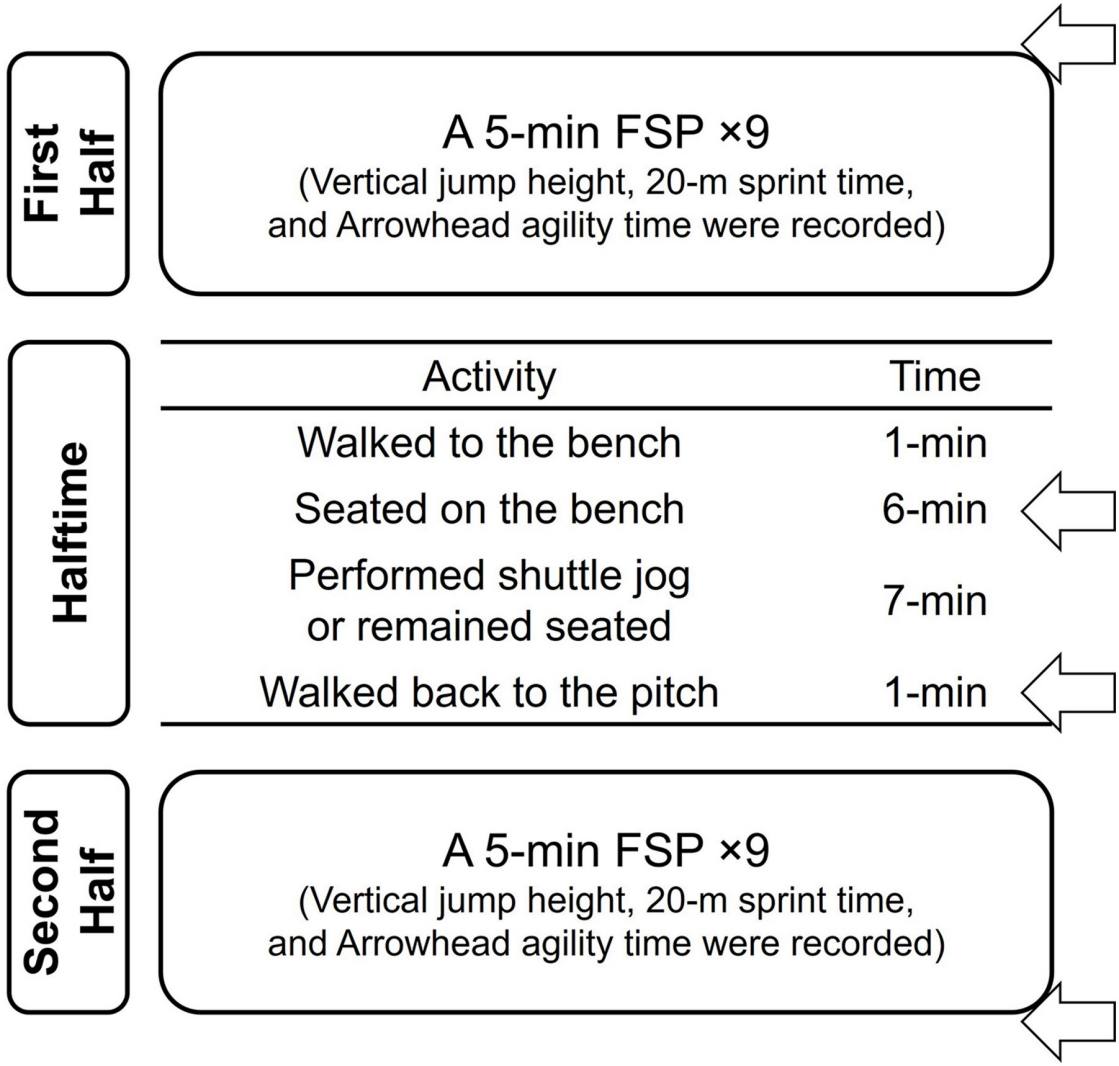
Testing procedures. Subjects were randomly experienced the condition of shuttle jog or seated rest each session. Heart rate was recorded throughout the experiment. The arrows indicate time points for lower-leg temperature measurements. FSP: football simulation protocol.

Upon arrival to the football pitch, testing procedures were instructed and written informed consent were obtained. Each session began with a self-directed 10- to 15-min warm-up (light jog and dynamic stretch). Subjects were equipped with the thermistor probes and heart rate monitor, the baseline lower-leg temperature and heart rate were recorded 1-min before the first half (Fig 2). After the thermistor probes were detached, subjects performed nine repetitions of the 5-min FSP (Fig 1) to represent the first and second halves. Once halftime begun, subjects walked to the bench (took 1-min), sat on the shaded bench (located in the sideline area), and rested for 6-min while consuming water (500 mL). During this time, temperature probes were attached again to the lower-leg. After the temperature data were taken, subjects randomly performed either condition (shuttle jog or seated rest) for the next 7-min at each session. Subjects performed a 20-m shuttle jog at a constant intensity (average 70% of heart rate maximum recorded during the first half) on a football pitch [8]. To maintain the intensity of the shuttle jog, we continuously monitored their heart rate and gave continuous verbal feedback (e.g., “slow down or a little faster”) to players. For the seated rest, subjects maintained the position (sitting on the bench) for another 7-min. During the last minute of halftime, lower-leg temperatures were taken again for both conditions. Subjects then completed another nine repetitions of the FSP for the second half (same activity as the first half). The temperature probes were attached again to the lower-leg immediately after the second half. Heart rate was recorded throughout the whole experiment. For the second session, subjects came back for another condition and went through the same experimental procedures.

### Endpoints

For the maximal vertical jump, subjects were instructed to place their feet on the pitch within a triangle area (Fig 1) and to vertically jump as high as they could using both legs. Take-off movements were self-selected and performed pre-stretch using their lower-extremity joints and both arms’ swinging. Two cameras were set up as 240 fps with 1/1000 shutter speed: One camera (C1 in Fig 1: 1-m away from the center of the jumping area and located 1-m high from the grass) videotaped subjects’ feet to determine flight time while the other camera (C2: 1-m away from the centre of the jumping area and located 20 cm high from the grass) videotaped subjects’ whole-body. Video clips from the two cameras were exported into a motion analysis software (Kinovea 0.8.15, Kinovea Org., France) and synchronised [19]. Flight time (t: ms) as counting the number of frames from take-off to landing was first calculated. Subjects’ last foot to take-off and the first foot to touch the ground were considered as take-off and landing, respectively. Flight time was then inserted into the previously established formula [h = (1000 × t^2^) × 1.22625 / 10000] [20] to calculate the maximal vertical jump height (h: cm). This countermovement vertical jump was performed twice in the FSP; thus, a total of six jumps were averaged to represent each time point. For the 20-m sprint, subjects were asked to run straight as fast as they could with a standing start position (50 cm away from the start line). Two pairs of infrared timing sensors (Brower Timing System, Salt Lake City, USA) were set up at the start and finish lines, located 20-m apart. This activity was performed twice in the FSP; thus, a total of six 20-m sprint times were averaged to represent each time point. Subjects started 50 cm away from the start line for the Arrowhead agility test. Arrowhead agility test started towards cone-A (placed 5-m away from the start line), moved to cone-B and cone-C, and sprinted to the finish line [21] (Fig 1). Timing systems were used as the start line and finish line to record the time taken to sprint the agility course. This activity was performed twice in the FSP; thus, a total of six Arrowhead agility test times were averaged to represent each time point.

**Fig 3:**
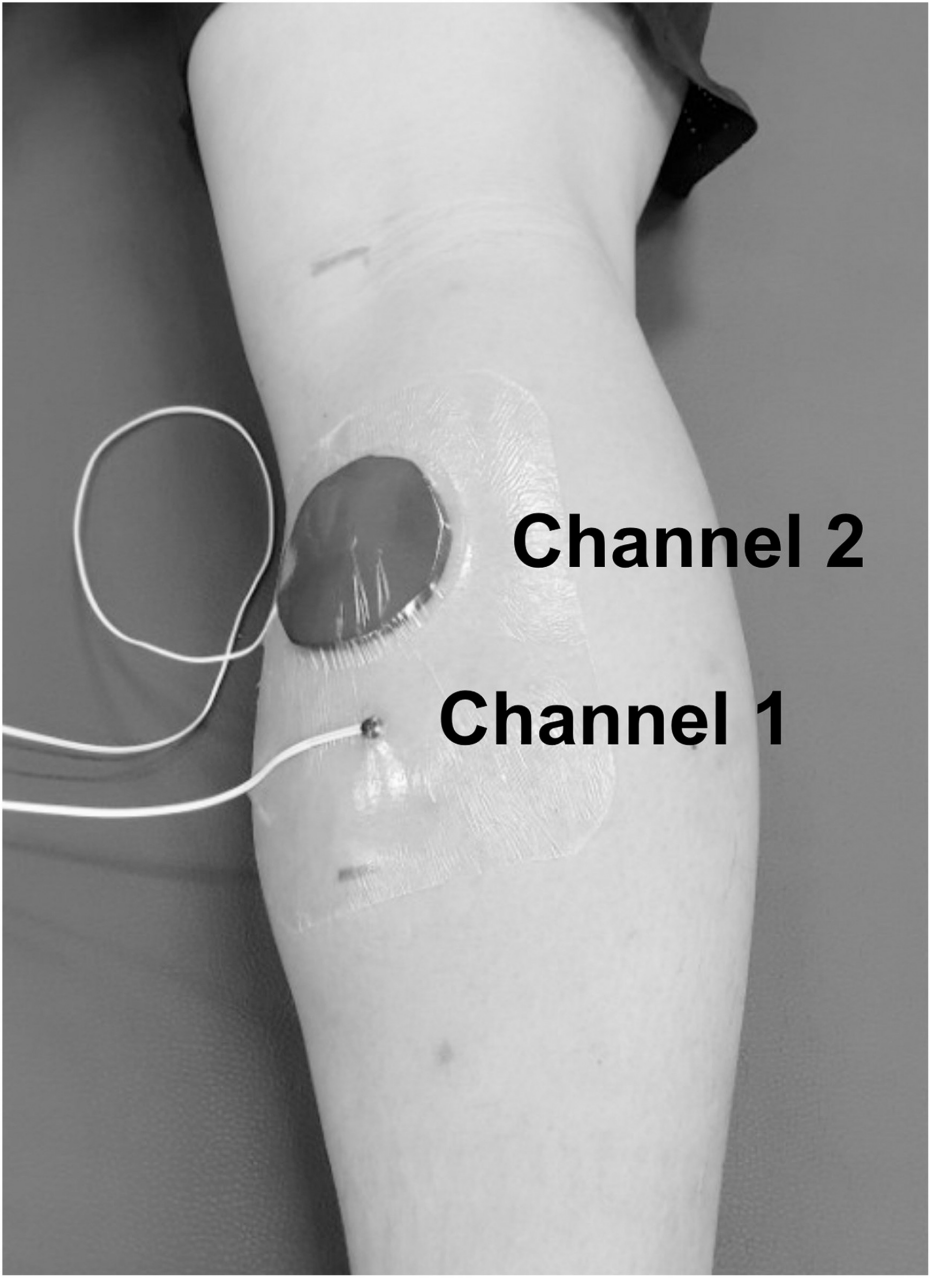
Lower-leg temperature measurements. The thermistor probes were attached to the lower-leg (Channel 1: skin temperature, Channel 2: muscle temperature).

To sample skin and muscle temperatures of the lower-leg, two thermistor probes, attached as two separate channels, to the digital thermometer logger (sampling rate: 60 Hz; NT logger, NKTC, Tokyo, Japan) were used. Each thermistor probe was attached to the gastrocnemius medialis. Two thermistor probes (connected to each channel) were attached to the middle of the gastrocnemius medialis muscle belly [22] in the dominant leg (the foot to kick a penalty shootout). Channel 1 and 2 of the probes were attached into the distal 1/3 (skin) and the proximal 1/3 (muscle). The thermistor probe (channel 1) for skin temperature measurement was secured with film dressing (Tegaderm Film, 3M, St, Paul, USA). The thermistor probe (channel 2) for the muscle temperature was covered by neoprene fabric and secured with the film dressing (Fig 3). This device has been validated [23] and shown high measurement reliability with the intraclass correlation coefficient of 0.93 [24]. These data were collected four times (before and after the first and second half: Fig 2). To record heart rate, a strap monitor (Polar H7, Polar Electro Oy, Kempele, Finland) was worn on the subjects’ chest. Once subjects’ demographic information (sex, height, and mass) was entered, the strap was wirelessly connected to a cellphone application (WearLink, sampling rate: 60 Hz). This device has been validated and shown high measurement reliability with the intraclass correlation coefficient of 0.80 to 0.86 [25]. Heart rate data in every 15-min interval (900 data points) were averaged to represent each time point.

### Statistical analysis

An a priori power analysis was performed based on the previous data about athletic performance. An expected mean difference in maximal vertical jump of 4.3 cm with a standard deviation of 6.3 cm [26] and in 20-m sprint time of 0.13 sec with a standard deviation of 0.18 sec [27] estimated that a minimum of 17 and 14 subjects were necessary, respectively (an alpha of 0.05 and a beta of 0.2). Therefore, a sample size in this study was determined as 17.

To test the condition effects over time in athletic performance, lower-leg temperature, and heart rate, we performed mixed model analysis of variance (random variable: subject; fixed variable: condition and time). The least significant difference was used for post hoc pairwise comparisons (SAS Institute Inc, Cary, USA, *p*<0.05 for all tests). If statistical differences appeared, we also calculated between-time effect sizes (ES) using the formula $\left[\text{ES}=[\bar{\text{X}}1-\bar{\text{X}}2]\;/\;\sigma \text{pooled}\right]$.

## Results and discussion

### Athletic performance

**Fig 4:**
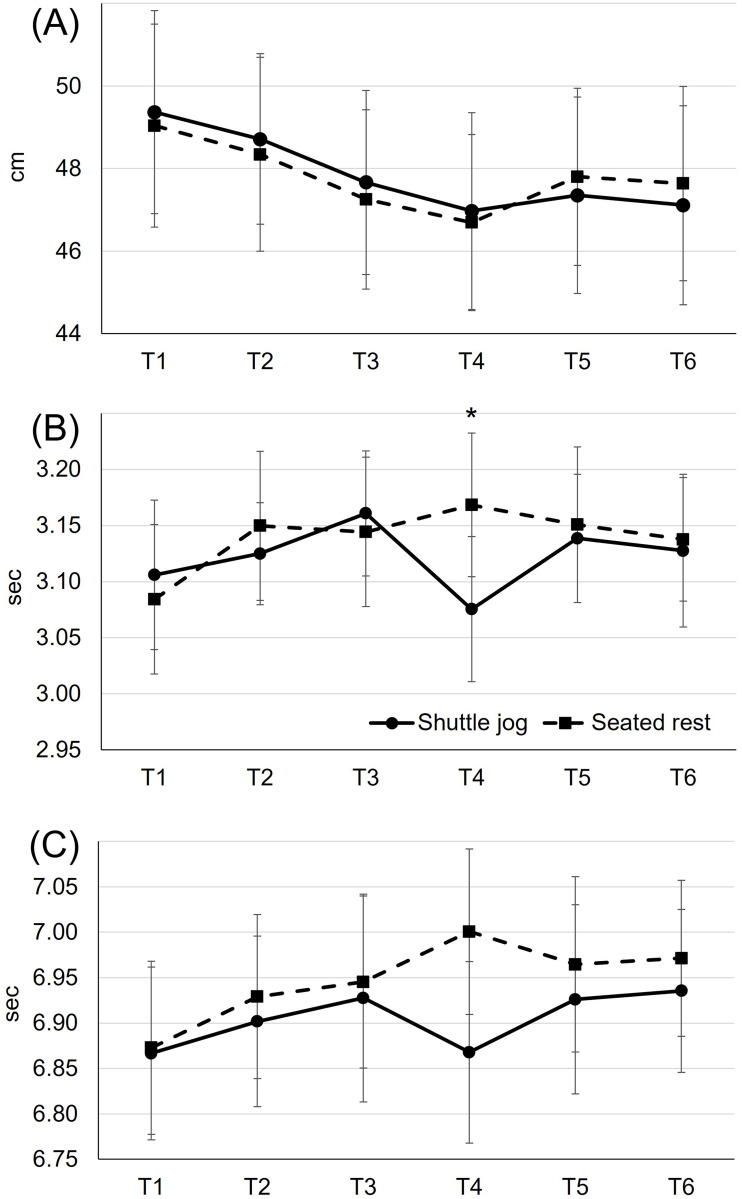
Changes in maximal vertical jump height (A), 20-m sprint time (B), and Arrowhead agility test time (C) over time. Values are means and the upper and lower bounds of 95% confidence intervals. * A difference between conditions during T4 (*p* = 0.002, 3.08 vs. 3.17 sec, 3%, ES = 0.68).

Maximal vertical jump height was not different (condition × time: F_5,187_ = 0.53, *p* = 0.75, η^2^ = 0.002; condition: F_1,187_ = 0.08, *p* = 0.78, η^2^<0.0001, Fig 4A). Regardless of condition (time: F_5,187_ = 8.62, *p*<0.0001, η^2^ = 0.03), subjects’ maximal vertical jump height was higher during T1 (49.2 cm) compared to T3 (47.5 cm, *p*<0.0001, 4% ES = 0.37), T4 (46.8 cm, *p*<0.0001, 5%, ES = 0.50), T5 (47.6 cm, *p*<0.0001, 3%, ES = 0.33), and T6 (47.4 cm, *p*<0.0001, 4%, ES = 0.37).

There was condition effect over time in 20-m sprint time (condition × time: F_5,187_ = 2.42, *p* = 0.04, η^2^ = 0.02; condition: F_1,187_ = 0.41, *p* = 0.53, η^2^ = 0.0007, Fig 4B). Specifically, subjects who performed the shuttle jog ran 0.09 sec faster (3.08 sec, *p* = 0.002, 3%, ES = 0.68), as compared with those who did the seated rest (3.17 sec) during the initial 15-min of the second half. Regardless of condition (time: F_5,187_ = 2.80, *p* = 0.02, η^2^ = 0.025), sprint time decrement was observed during T3 (3.16 sec), as compared to T1 (3.10 sec, *p*<0.0001, 2%, ES = 0.44) the last 15-min of the first half.

Arrowhead agility test time was not different (condition × time: F_5,187_ = 1.25, *p* = 0.29, η^2^ = 0.011, Fig 4C). Regardless of time (condition: F_1,187_ = 5.12, *p* = 0.03, η^2^ = 0.009), subjects who performed the shuttle jog ran 0.04 sec faster than those who did the seated rest (6.91 vs. 6.95 sec, ES = 0.18). Regardless of condition (time effect: F_5,187_ = 2.37, *p* = 0.04, η^2^ = 0.02), subjects’ Arrowhead agility test time during T1 (6.87 sec) was faster, compared to that during T6 (6.95 sec, *p*<0.0001, 1%, ES = 0.39).

### Lower-leg temperature

**Fig 5:**
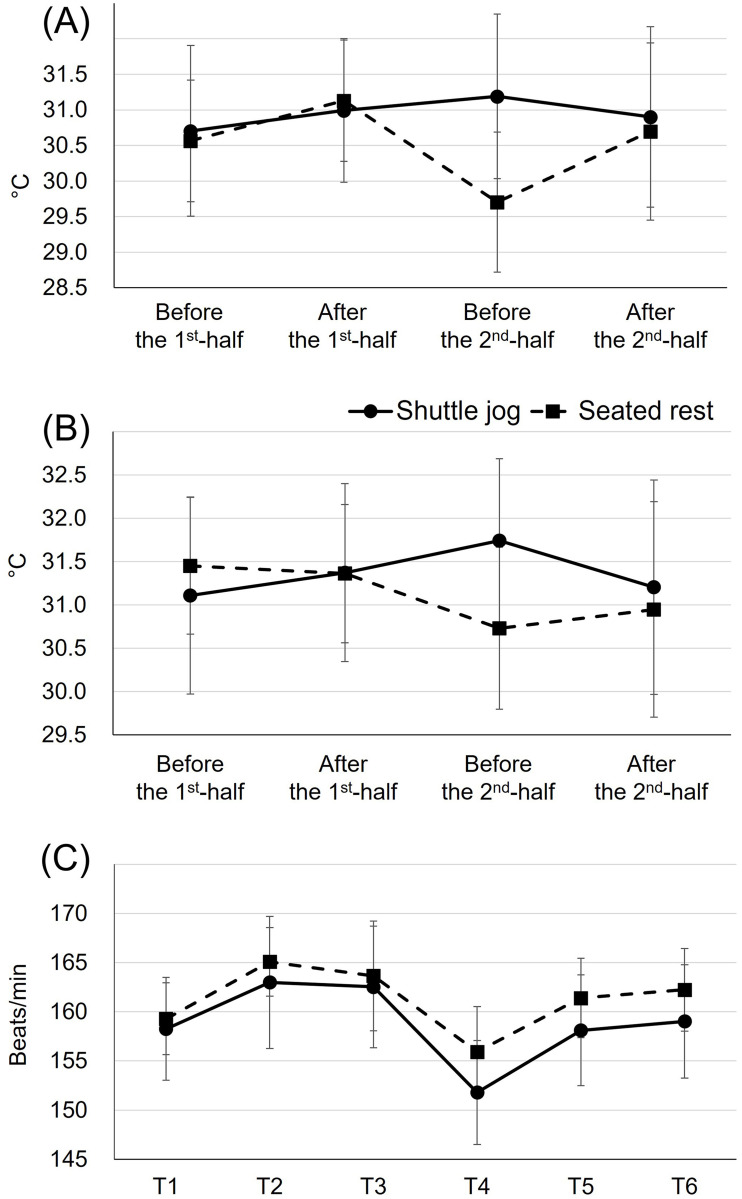
Changes in lower-leg skin (A) and muscle (B) temperature, and heart rate (C) over time. Values are means and the upper and lower bounds of 95% confidence intervals.

There was no condition effect over time in the skin (condition × time: F_3,119_ = 1.40, *p* = 0.25, η^2^ = 0.020; condition: F_1,119_ = 1.49, *p* = 0.22, η^2^ = 0.007; time: F_3,119_ = 0.67, *p* = 0.57, η^2^ = 0.01, Fig 5A) and muscle (condition × time: F_3,119_ = 1.08, *p* = 0.36, η^2^ = 0.013; condition: F_1,119_ = 0.72, *p* = 0.40, η^2^ = 0.003; time: F_3,119_ = 0.20, *p* = 0.90, η^2^ = 0.002, Fig 5B) temperature of the lower-leg.

### Heart rate

The average heart rate during the whole match was recorded as 160 ± 11 bpm. We did not observe condition effects throughout any time point on heart rate (condition × time: F_5,187_ = 0.31, *p* = 0.91, η^2^ = 0.003, Fig 5C). Regardless of time (condition: F_1,187_ = 7.09, *p* = 0.008, η^2^ = 0.013), subjects with the shuttle jog showed 2% less heart rate than those with the seated rest (159 vs. 161 bpm, ES = 0.22). Regardless of condition (time: F_5,187_ = 10.05, *p*<0.0001, η^2^ = 0.086), subjects’ heart rate was lower during the initial 15-min of the second half (T4: 154 bpm), as compared with other time points (T1: 159 bpm, *p* = 0.003, 3%, ES = 0.48; T2: 164 bpm, *p*<0.0001, 6%, ES = 0.91; T3: 163 bpm, *p*<0.0001, 6%, ES = 0.79; T5: 160 bpm, *p* = 0.0003, 4%, ES = 0.55; T6: 161 bpm, *p*<0.0001, 4%, ES = 0.62).

We were interested in studying the effect of a 7-min halftime shuttle jog on subsequent lower-leg temperature and performance change in the second half as compared with a seated rest. Our hypotheses were partly accepted as subjects who performed the halftime shuttle jog maintained the sprint performance during the initial 15-min period of the second half when compared with those who did the seated rest. Our data confirmed that a decremental effect of the static rest on sprinting performance can be attenuated by a halftime warm-up. The limitations from previous data such as inter-individual variability [8, 13], non-football-specific activity [10, 15], and small sample sizes [10, 12, 14, 15] have been overcome by the results of our study. In connection with the lower-leg muscle temperature, a 3% difference in sprint performance (3.08 vs. 3.17 sec) during T4 is reinforced by a previous suggestion that a 1°C difference in muscle temperature may lead to a variation of performance capacity ranged between 2% and 5% [28, 29].

Our data on sprint performance change in both conditions during T4 has practical implications. Specifically, the difference in the speed of each performance based on the records was calculated as 0.18 m/s (6.49 vs. 6.31 m/s), which would result in a difference in the distance covered in a given amount of time. For example, a player with the halftime warm-up would be approximately 0.4-m ahead of a player with the seated rest, when running 2 sec (13.0-m vs. 12.6-m). Assuming all other personal and environmental conditions are similar, this could be the main scene of the match when considering the importance of sprinting in football. Since > 90% of sprints were finished within 5 sec [30] and receiving a pass (travelled > 10-m) was the most common attacking option for goal scoring [31] in actual football matches, our estimation in sprint distance was practically meaningful.

The difference of lower-leg muscle temperature between conditions during the initial 15-min showed a 1.0°C, which was not statistically different. Previously a higher quadriceps muscle temperature (1.2°C [8] and 1.0°C [14]) after a halftime shuttle jog [8] or agility exercise [14] was reported, as compared with the seated rest. These temperature differences led to a difference in athletic performance in which subjects with the halftime jog [8] or agility exercise [14] ran 0.20 sec [8] or 0.10 sec faster in 30-m or 10-m sprints [14], respectively. The linear relationship between rate of torque development and muscle temperature was also reported in football players [32]. Based on the previous data [8, 14, 33], a muscle temperature of 1.0°C seems to be a sufficient amount for performance change. The measurement using the insulation disk technique [34] allowed to estimate the tissue temperature at an approximate depth of 2.2 cm from the thermistor probe (Fig 3). Additionally, the lower-leg has a smaller muscle mass and distally located than the upper leg, the whole leg temperature was probably 1.0°C or higher in subjects who performed the halftime jog. Therefore, a 0.09 sec faster sprint time during T4 could have been, at least partially, involved with the prevention effect of halftime jog on the temperature reduction associated with the seated rest. While the lower-leg muscle temperature for the halftime jog during T4 was recorded as 31.7°C, we still do not know if this is an optimal temperature for sprint performance. Either a lower or elevated muscle temperature could impair muscle function [35], future studies should attempt to determine the ideal muscle temperature in relation to the given environmental conditions.

Due to the intermittent nature of football, various movements are performed at different intensities. The FSP [3] used in our study provided a similar situation of the intermittent characteristics of a real football match. For example, our subjects’ sprint performance did not decline during the last 30-min of the second half (T4 and T5) although fatigue and dehydration could have been occurred towards to the end of match [36]. A previous report on the effects of fatigue on football kick-related performance also showed that the ground reaction force and joint kinematics during instep kicks were not altered by fatigue during a 90-min intermittent exercises [37]. Additionally, a previous study reported that 70% of movements in a common adult football match was low-intensity (e.g., jogging or walking) [38]). The distance covered by high-intensity activities (e.g., 20-m sprint and Arrowhead agility test) in the 5-min FSP was 65-m, which is about 28% of the total distance covered (235-m) in our study. While the data under the control condition (e.g., seated rest) in our study mimics a typical football match, those under the halftime jog condition could, therefore, be applied to real football.

**Fig 6:**
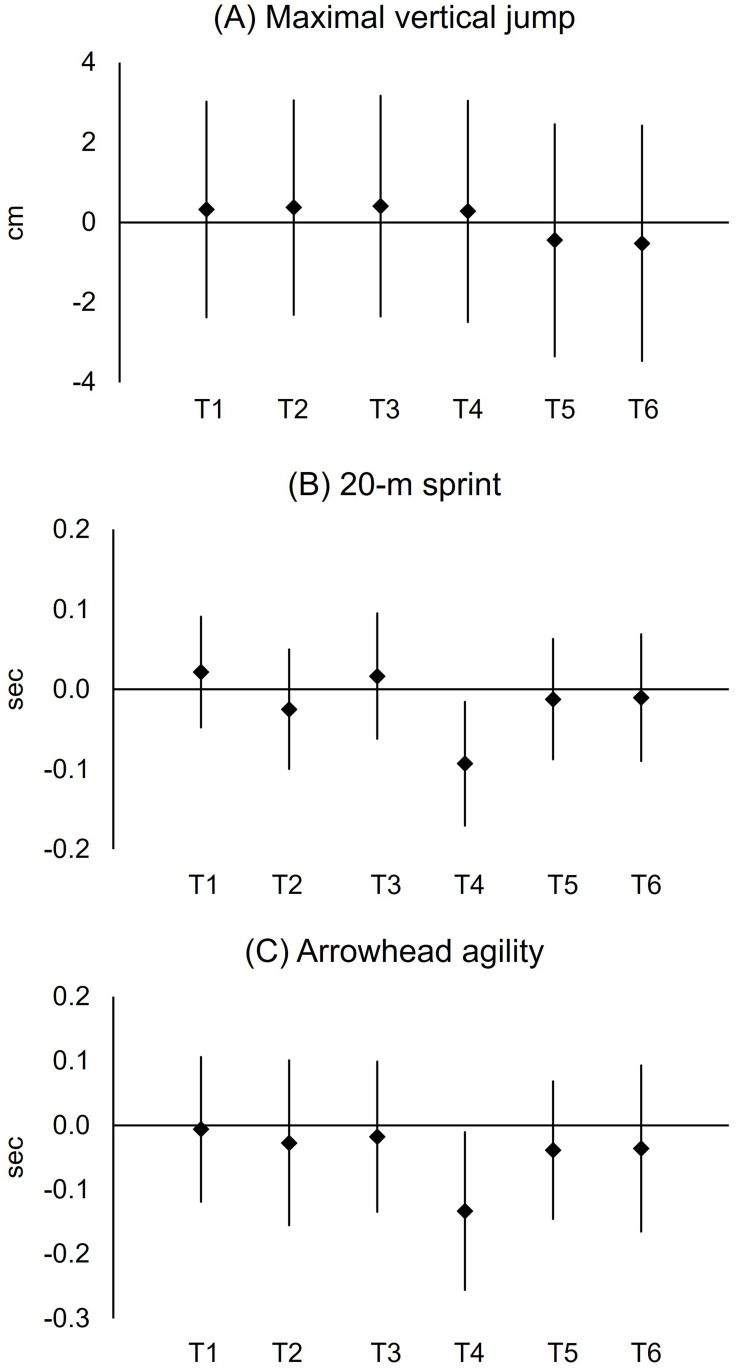
The 95% confidence intervals for difference in mean (maximal vertical jump height: A; 20-m sprint time: B; Arrowhead agility test time: C).

The halftime jog was not effective in preventing performance hinderance in jump height and agility time associated with the seated rest. Contrarily, a couple of previous studies [13, 14] reported a halftime warm-up as an effective strategy to prevent the decrements in jump performance. The discrepancy between our results and those in previous studies [13, 14] could be explained by the several different testing procedures, including simulation protocol (non-football-specific [14]), measurement time point (single measurements at 46-min [13, 14] and 60-min [14]), jump height assessment techniques (force plate [14] and motion capture system [13]), and environmental conditions (indoor laboratory [14] and 5–6°C with 84–87% humidity [13]). Additionally, the reported difference in jump heights between conditions (warm-up vs. seated rest during halftime) was ranged between 1 [13] and 2 cm [14], which was practically meaningless. In combination with the previous data [13, 14], our results suggest that the magnitude of deterioration in athletic performance due to muscle temperature reduction may be related to the frequency of the sport specific activities. Since running (17%) was a more frequent activity than jumping (4%) in football [39], decrements in sprint performance could have been larger than jump performance. This is indirectly supported by our data of athletic performance during T4 that the associated 95% confidence intervals of mean difference in vertical jump (Fig 6A) crosses zero while running activities (Fig 6B & 6C) does not.

Heart rate was secondarily analysed to back up any change in dependent measurements. We observed a condition effect that subjects with the halftime jog (159 bpm) showed 2 bpm less heart rate than subjects with the seated rest (161 bpm, ES = 0.22) in the second half. These results in heart rate values were very similar to those in actual football matches (halftime jog: 157 bpm; seated rest: 161 bpm) [13], indicating the applicability of the FSP used in our study. Change in the cardiopulmonary system due to training or detraining took at least 6 weeks [40], supposing the amount of stroke volume and cardiac output in subjects in our study was not different between the sessions. Therefore, our observation in less heart rate in subjects with the halftime jog could be explained by circulatory efficiency [41]. Under the autonomic regulation, the cardiovascular and hemodynamic adjustments occur to adequately deliver oxygen supply to the working muscles (e.g., lower-extremity) when exercising [42]. We believe that subjects who performed the halftime jog (average heart rate: 128 bpm) led to more active hemodynamics (e.g., exchanging nutrients and wastes) in the lower-extremity when compared with players who did the seated rest. Since the same workloads (the 5-min FSP ×9) to both conditions were given, the lower-extremity with a worse circulatory efficiency (e.g., seated rest condition) required a continuous blood supply to accommodate for the energy requirements. As a result, subjects with the halftime jog showed less heart rate.

Our study has several limitations. First, the data were collected under the environmental conditions (air temperature: 17.5°C, relative humidity 40%, and pitch condition: natural grass) on a specific population (collegiate male footballers with an athletic career of 10 years), which should be considered when generalising the results. In connection with the weather condition, a difference in core temperature between conditions should have certainly had an impact on athletic performance [43]. It should also be noted that the jog intensity was guided by the research assistant, not self-determined by the player. In case of under- or over-pacing, we recommend coaches and players practice getting used to the optimal intensity of the halftime jog.

## Conclusions

Our data demonstrated potential benefits of 7-min halftime jog on sprint performance during the initial 15-min of the second half. Our results are meaningful that the limitations of the current existing data [8, 10, 12–15] have been overcome by obtaining data from a 90-min simulated football match performed by a larger number of footballers. We recommend performing a moderate-intensity aerobic exercise (70% of the maximum heart rate) during halftime to avoid the negative effects associated with the sedentary halftime period.

## Supporting information

S1 Data (XLSX)
